# END-OF-LIFE EXPERIENCES AND CARE NEEDS IN THE LIVES OF OLDER LGBT PEOPLE

**DOI:** 10.1093/geroni/igz038.2325

**Published:** 2019-11-08

**Authors:** Kathryn Almack

**Affiliations:** School of Health and Social Work University of Hertfordshire, Hatfield, England, United Kingdom

## Abstract

The socio-cultural and legal position of LGBT citizens varies across nations. However, even in the most liberal countries, an historical legacy of stigma impacts on older LGBT people’s access to care. This paper draws upon the qualitative strand of a two-year UK project exploring the end of life experiences and care needs of older LGBT people. (N = 60 in-depth interviews with LGBT participants aged 60+). Findings highlight that the majority of respondents reported ways in which they manage their personal networks to minimize any vulnerability to discrimination. In planning or needing end-of-life-care, respondents identify new ‘layers’ of decisions about disclosing or hiding their sexual or gender orientation; informed by past experiences and fears about discrimination or exclusion from service providers. In conclusion, older LGBT people’s histories and a legacy of stigma have ongoing profound influences on the means of support available to them at the end of life.

